# Development of a predictive model for the risk of microalbuminuria: comparison of 2 machine learning algorithms

**DOI:** 10.1007/s40200-024-01440-4

**Published:** 2024-05-31

**Authors:** Wenyan Long, Xiaohua Wang, Liqin Lu, Zhengang Wei, Jijin Yang

**Affiliations:** 1 The Affiliated Hospital of Zunyi Medical University, Zunyi, 563099 China; 2 School of Medical Information Engineering, ZunyiMedical University, Zunyi, 563006 China

**Keywords:** Predictive models, Diabetes mellitus, Microalbuminuria, Random Forest, BP neural network, Machine learning, Factors, SHAP

## Abstract

**Purpose:**

To identify the independent risk variables that contribute to the emergence of microalbuminuria(MAU) in type 2 diabetes mellitus(T2DM), to develop two different prediction models, and to show the order of importance of the factors in the better prediction model combined with a SHAP(Shapley Additive exPlanations) plot.

**Methods:**

Retrospective analysis of data from 981 patients with T2DM from March 2021 to March 2023. This dataset included socio-demographic characteristics, disease attributes, and clinical biochemical indicators. After preprocessing and variable screening, the dataset was randomly divided into training and testing sets at a 7:3 ratio. To address class imbalance, the Synthetic Minority Oversampling Technique (SMOTE) was applied to balance the training set. Subsequently, prediction models for MAU were constructed using two algorithms: Random Forest and BP neural network. The performance of these models was evaluated using k-fold cross-validation (k = 5), and metrics such as the area under the ROC curve (AUC), accuracy, precision, recall, specificity, and F1 score were utilized for assessment.

**Results:**

The final variables selected through multifactorial logistic regression analysis were age, BMI, stroke, diabetic retinopathy(DR), diabetic peripheral vascular disease (DPVD), 25 hydroxyvitamin D (25(OH)D), LDL cholesterol, neutrophil-to-lymphocyte ratio (NLR), and glycated haemoglobin (HbA1c) were used to construct the risk prediction models of Random Forest and BP neural network, respectively, and the Random Forest model demonstrated superior overall performance (AUC = 0.87, Accuracy = 0.80, Precision = 0.79, Recall = 0.84, Specificity = 0.76, F1 Score = 0.81). The SHAP feature matrix plot revealed that HbA1c, NLR, and 25(OH)D were the three most significant factors in predicting the development of MAU in T2DM, with 25(OH)D acting as an independent protective factor.

**Conclusion:**

Effective identification of MAU in T2DM, therapeutic strategies for controllable high-risk factors, and prevention or delay of diabetic kidney disease(DKD) can all be achieved with the help of the risk prediction model developed in this study.

## Introduction

The International Diabetes Federation (IDF) has recently unveiled a new global diabetes map [1] indicating that approximately 537 million adults worldwide are afflicted with diabetes. Furthermore, projections suggest a 16% increase in the global diabetic population from 2019 to 2021. Of particular concern is the outlook for Chinese diabetic patients, with estimates anticipating numbers to escalate to 164 million in 2030 and 175 million in 2045. DKD is a prevalent microvascular complication associated with diabetes, impacting around 20–40% of individuals diagnosed with diabetes. Key clinical features of DKD include sustained elevation in albumin excretion in urine and a gradual decline in glomerular filtration rate (GFR). DKD has emerged as the primary cause of end-stage renal disease (ESRD) [2], posing significant implications for patient well-being, substantial financial burdens, and potential reduction in life expectancy.

According to the latest Chinese DKD clinical diagnosis and treatment guidelines [3], the progression of DKD involves increased glomerular filtration rate, MAU, macroalbuminuria, and ultimately, renal failure. Notably, the MAU stage represents the earliest clinically diagnosable phase of DKD. MAU, being the initial stage of DKD, has been observed to advance to the clinical stage in 20-40% of untreated patients, characterized by severe albuminuria. At this juncture, the progression towards renal failure is significantly accelerated [4]. Numerous studies [5–7] have substantiated that comprehensive interventions, encompassing control of blood glucose, blood pressure, and weight loss, can transform the stage of massive albuminuria to normoalbuminuria or urinary albumin-negativity, thereby enhancing patients’ quality of life and influencing renal outcomes positively. Importantly, MAU is considered reversible. Hence, timely screening and identification of MAU, coupled with effective intervention, can potentially prevent or reverse albuminuria, crucial for delaying the onset of cardiovascular disease endpoint risk events.

Recognizing the significance of MAU in predicting cardiovascular disease risk, this study aimed to achieve two primary objectives: (1) to compare two machine learning algorithms for the creation of a risk prediction model for MAU in patients with T2DM, and (2) to integrate the SHAP framework model to visually illustrate the significance of independent factors associated with MAU in T2DM patients. The overarching goal is to contribute insights for personalized medical management and health promotion strategies aimed at mitigating cardiovascular disease risks.

## Methods

### Study design and population

This retrospective study focused on hospitalized patients, specifically involving a random selection of 981 individuals diagnosed with T2DM who were admitted to the Department of Endocrinology and Metabolic Diseases at the Affiliated Hospital of Zunyi Medical University and the data collection period spanned from March 2021 to March 2023.

Diagnostic criteria for T2DM were established according to the 1999 World Health Organization (WHO) criteria [8]. For the diagnosis of DKD and MAU, the study adhered to the diagnostic guidelines outlined in the Chinese Guidelines for Clinical Diagnosis and Treatment of DKD [3]. The study subjects were categorized based on their urinary albumin excretion rate (UAER). Specifically, individuals with T2DM exhibiting a UAER ranging from 30 to 300 mg/24 h were assigned to the MAU group (*n* = 333). In contrast, those with T2DM and a UAER less than 30 mg/24 h were classified into the DM group (*n* = 648).

Inclusion criteria: (1) Age ≥ 18 years; (2) T2DM patients.

Exclusion criteria: (1) diabetic ketoacidosis; (2) gestational diabetes mellitus and other special types of diabetes mellitus; (3) hyperglycaemic hyperosmolar state or severe and recurrent hypoglycaemic events in the past 3 months; (4) severe renal or hepatic malfunction, psychological illnesses, or a history of cancerous tumors; (5) renal injury due to other diseases.

### Estimating the required sample size

This study adhered to the EPV (Events Per Variable) principle for determining the sample size of the predictive model, as outlined in the literature [9]. With 15 independent variables entered into the logistic regression analysis, and considering an estimated incidence of MAU in patients with T2DM ranging from 20–40% [10]—taking the midpoint of 30%—and factoring in a 10% attrition rate, the minimum required sample size for the predictive model modeling group in this study was calculated as follows:

$$ N=\frac{\text{n}\text{u}\text{m}\text{b}\text{e}\text{r} \text{o}\text{f} \text{i}\text{n}\text{d}\text{e}\text{p}\text{e}\text{n}\text{d}\text{e}\text{n}\text{t} \text{v}\text{a}\text{r}\text{i}\text{a}\text{b}\text{l}\text{e}\text{s} \times 10}{\text{i}\text{n}\text{c}\text{i}\text{d}\text{e}\text{n}\text{c}\text{e}}\times \left(1+10\text{\%}\right)$$

The modeling group comprised 2/3 of the total sample size, and the validation group constituted the remaining 1/3. The minimum sample size for the validation group was determined to be *N* = 275 cases, resulting in a total minimum sample size of *N* = 825 cases. The final inclusion of 981 cases in this study exceeded the sample size requirement. All participants were randomly divided into a training set (687 cases) and a test set (294 cases) in a 7:3 ratio. The training set was utilized for model construction, while the test set was employed for model validation, ensuring a robust assessment of the predictive model’s performance.

### Data extraction and clinical indicator measurements

The electronic medical record system within the hospital served as the primary data source, encompassing a comprehensive set of 36 candidate variables for predictive modeling. These variables were thoughtfully selected based on systematic meta-analysis and consultation with clinical care specialists and medical experts. The candidate variables were further classified into three distinct categories: socio-demographic characteristics, disease characteristics, and clinical biochemical indicators.

### Socio-demographic characteristics

Gender.

Age.

Days in hospital.

Number of hospitalizations.

Residence.

Education level.

Smoking.

Drinking.

Systolic blood pressure (SBP).

Diastolic blood pressure (DBP).

Body mass index (BMI).

### Disease characteristics

Diabetes duration.

Family history of diabetes.

Glucose-lowering regimen (oral hypoglycemic agents, insulin therapy).

Past medical history (hypertension, stroke).

Diabetic complications (diabetic peripheral neuropathy (DPN), diabetic retinopathy (DR), Diabetic peripheral vascular disease (DPVD)).

### Clinical biochemical indicators

Glycated hemoglobin (HbA1c).

Fasting blood glucose (FBG).

Total cholesterol (TC).

Triacylglycerol (TG).

High-density lipoprotein cholesterol (HDL-C).

Low-density lipoprotein cholesterol (LDL-C).

Albumin (ALB).

Blood creatinine (Scr).

Uric acid (UA).

Urea nitrogen (BUN).

Serum C-peptide.

2-hour postprandial serum C-peptide.

Neutrophil-to-lymphocyte ratio (NLR).

Platelet-to-lymphocyte ratio (PLR).

Mean platelet volume (MPV).

Serum 25-hydroxyvitamin D (25(OH)D).

These variables collectively constitute a comprehensive dataset that captures socio-demographic, disease-related, and clinical biochemical aspects, offering a robust foundation for the predictive modeling undertaken in this study.

The BMI was calculated by dividing an individual’s weight by the square of their height, expressed in kilograms per square meter (kg/m^2^). For individuals with a history of hypertension or those diagnosed with hypertension during hospital admission, the diagnostic criteria outlined in the latest practice guidelines were followed [11]. Previous stroke history, whether ischemic or hemorrhagic, was also taken into consideration. The diagnosis of DR was established through clinical diagnosis and fundus photography. DPN was diagnosed based on the patient’s medical history, physical examination, and electromyography. DPVD was identified through clinical evaluation and the detection of vascular ultrasound abnormalities.

Upon admission, all patients underwent a fasting period of over 8 h overnight. Subsequently, 5 ml of venous blood was drawn from the elbow in the early morning of the following day, while patients remained in a fasted state. Various biochemical indicators were analyzed using specific methods:

FBG was measured by the hexokinase method. HbA1c was detected by the high-pressure liquid chromatography method.TC, TG, HDL-C, LDL-C, ALB, Scr, BUN, Uric Acid UA, and Serum C-peptides were analyzed by the Olympus (AU2700) automatic analyzer. Serum C-peptide 2 h after a meal, platelet count, neutrophils, and lymphocytes were detected using a hematology analyzer.MPV was determined using a hematology analyzer. 25(OH)D was detected by electrochemiluminescence (reagents were purchased from Roche, Germany).MAU was determined using the Coulter Beckman AU5421 Automatic Specific Protein Analyzer, employing immunoscattering turbidimetry and supporting reagents. These meticulous diagnostic and laboratory procedures ensured comprehensive and accurate data collection for the study.

### Data pre-processing

Data sets containing more than 20% missing values were excluded from the analysis. For the remaining missing values, imputation was performed based on the type of variable: the mean or median was used for numeric variables, while the plurality method was applied for categorical variables.This approach ensured a robust analysis while accounting for any potential gaps in the dataset.Given that the dataset primarily consists of numerical variables with widely varying ranges, we implemented standardization on the training set using normalization techniques to ensure model accuracy. The same scaling was then applied to the test set for consistency.

### Statistical analysis

Data processing and statistical analysis were conducted using IBM SPSS (version 29.0). In analyzing the factors influencing the occurrence of MAU in patients with T2DM, it was observed that none of the continuous variables followed a normal distribution. Therefore, between-group comparisons were conducted using the Mann-Whitney U test, with results expressed as M (P_25_, P_75_). Categorical information was presented as n (%), and between-group comparisons were performed using the chi-square (*χ*^*2*^) test. Additionally, hierarchical information was compared using the Kruskal-Wallis H test. A significance level of *P* < 0.05 was deemed statistically significant.

### Model development

**Fig. 1:**
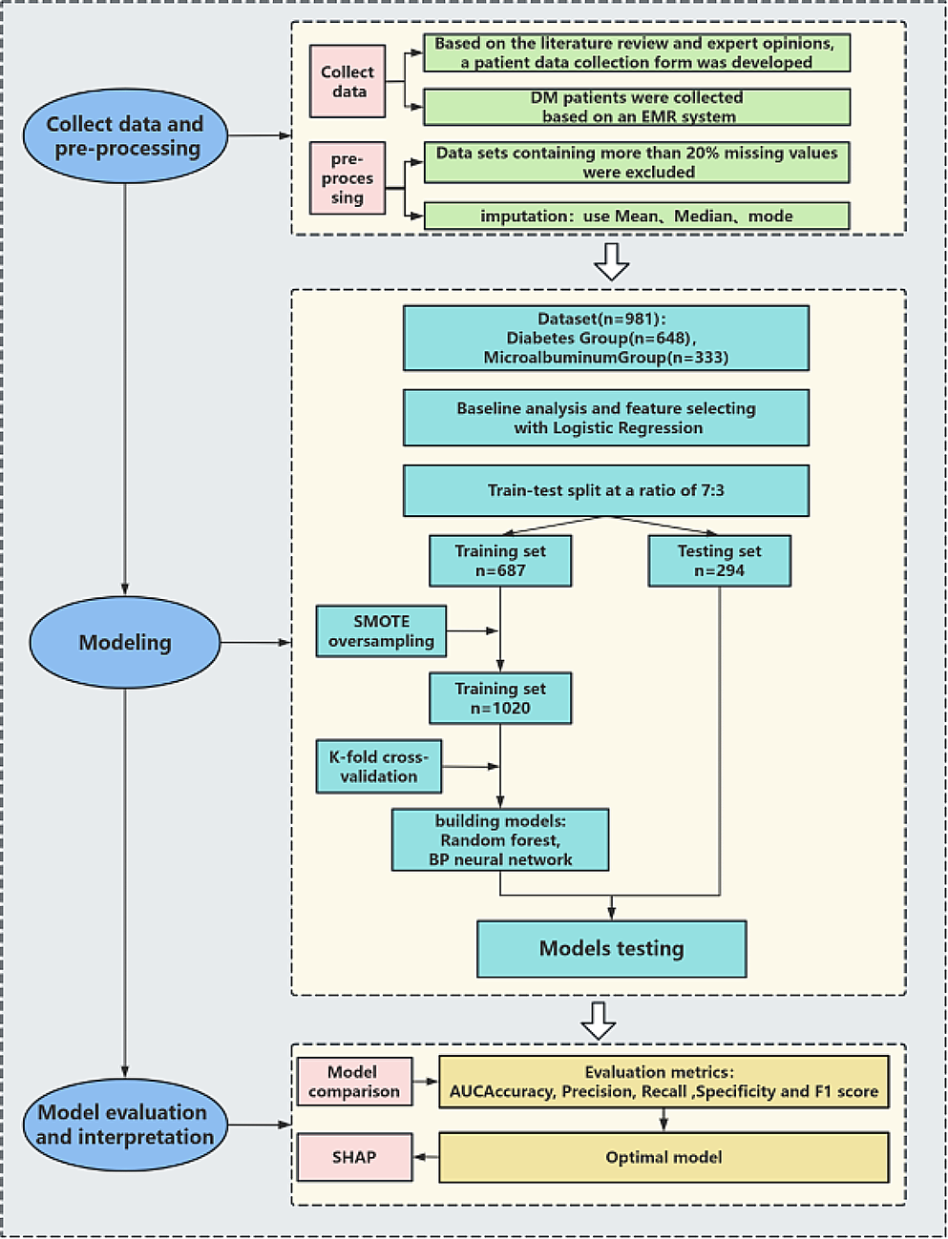
Establishment of a Forecasting Framework. SMOTE, Synthetic Minority Oversampling Technique, SHAP, Shapley Additive exPlanations

The risk prediction model was constructed using MATLAB software (version 2016b). The candidates for the prediction model underwent analysis through logistic regression. The independent variables identified through screening were then input into both Random Forest and BP neural network, respectively. The exact steps for building the prediction model in this study are outlined in Fig. 1.

The modeling process randomly divides the original dataset into a training set (70%) and a test set (30%), maintaining a balance of positive and negative samples. It’s worth noting that patient data in medicine often exhibit class imbalance, where general machine learning algorithms may favor predicting the majority class (usually samples) [12]. In this study, the dataset comprises 333 MAU patients and 648 MAU-negative patients. To address this data imbalance, we applied the Synthetic Minority Over-sampling Technique (SMOTE) [13] to the training data. Additionally, to mitigate randomness, the training set underwent cross-validation using the k-fold method (k = 5). This involves reserving one-fifth of the training set for testing, with the remaining four-fifths used for training iteratively. Model performance was evaluated using metrics such as the Area Under the ROC Curve (AUC), accuracy, precision, recall, specificity and F1 score. The average values of these metrics were calculated based on cross-validation.

### Model interpretation

Effective model interpretation is crucial for clinical predictive models, and integrating interpretable machine learning algorithms enhances transparency and interpretability in clinical decision-making. In recent years, the SHAP method has emerged as a recognized tool for interpreting predictive models. SHAP is an algorithm designed to evaluate the contribution of multiple factors towards an outcome. This algorithm assigns weights to each feature in the model, calculating and ranking the influence of each feature on the outcome [14]. The SHAP model employs Shapley values to visualize the results of the predictive model in a SHAP plot.

## Results

### Characteristics of the study population

A total of 981 patients with T2DM were included in this study. The sociodemographic features, disease characteristics, and clinical biochemical indicators of the study participants are summarized in Table 1. All the patients were categorized into MAU and DM groups. There was no statistically significant difference between the two groups concerning gender, days in the hospital, number of hospitalizations, residence, education level, smoking, drinking, DBP, family history of diabetes, insulin therapy, oral hypoglycemic agents, TG, TC, HDL-C, serum C-peptide, 2-hour serum C-peptide, ALB, Scr, UA, BUN, MPV. The two groups showed statistical significance in terms of age, SBP, DBP, BMI, duration of diabetes mellitus, history of hypertension, stroke, DR, DPN, DPVD, HbA1c, FBG, LDL-C, NLR, PLR, and 25(OH)D (*P* < 0.05).

**Table 1:** Baseline Characteristics of Patients with T2DM

Characteristics		Overall(*n* = 981)	MAU Group(*n* = 333)	DM Group(*n* = 648)	χ²/Z	*P*-value
Socio-demographic						
Gender(%)	Male	522.00(53.20)	177.00(53.20)	345.00(53.20)	0.001	0.979
	Female	459.00(46.80)	156.00(46.80)	303.00(46.80)		
Age(years)		58.00(51.00, 65.00)	59.00(52.00, 67.00)	57.00(50.00, 65.00)	-2.706	0.007**
Days in hospital		9.00(7.00, 11.00)	9.00(7.00, 11.00)	9.00(7.00, 11.00)	-0.311	0.755
Number of hospitalisations		2.00(1.00, 3.00)	2.00(1.00, 3.50)	2.00(1.00, 3.00)	-1.544	0.122
Residence(%)	Town	626.00(63.08)	207.00(62.20)	419.00(64.70)	0.595	0.441
	Countryside	355.00(36.20)	126.00(37.80)	229.00(35.30)		
Educational level(%)	Primary and below	430.00(43.80)	275.00(42.40)	155.00(46.5.0)	3.305	0.508
	Junior	253.00(25.80)	170.00(26.50)	83.00(24.90)		
	Senior	81.00(8.30)	54.00(8.30)	27.00(8.10)		
	post-secondary	107.00(10.90)	69.00(10.60)	38.00(11.40)		
	Undergraduate and above	110.00(11.20)	80.00(12.30)	30.00(9.00)		
Smoking(%)	Yes	324.00(33.00)	113.00(33.90)	211.00(32.60)	0.187	0.665
	No	657.00(67.00)	220.00(66.10)	437.00(67.40)		
Drinking(%)	Yes	289.00(29.50)	94.00(28.20)	195.00(30.10)	0.368	0.544
	No	692.00(70.50)	239.00(71.80)	453.00(69.90)		
SBP(mmHg)		130.00 (120.00, 139.00)	131.00 (122.00, 141.50)	128.00 (120.00, 138.00)	-3.388	0.001**
DBP(mmHg)		79.00(74.00, 86.50)	80.00(75.00, 88.00)	79.00(73.00, 86.00)	-1.863	0.062
BMI(kg/m2)		26.40(23.90, 27.90)	26.60(24.90, 28.60)	25.80(23.60, 27.80)	-4.399	0.000**
Diabetes Duration(years)		8.00(4.00, 12.00)	10.00 (4.00, 13.00)	8.00(3.00, 12.00)	-2.003	0.045*
Family history of diabetes(%)	Yes	40.00(4.10)	13.00(3.90)	27.00(4.20)	0.039	0.844
	No	941.00(95.90)	320.00(96.10)	621.00(95.80)		
Characteristics		Overall(*n* = 981)	MAU Group(*n* = 333)	DM Group(*n* = 648)	χ²/Z	*P*-value
insulin therapy(%)	Yes	614.00(62.60)	209.00(62.80)	405.00(62.50)	0.006	0.936
	No	367.00(37.40)	124.00(37.20)	243.00(37.50)		
Oral hypoglycemic agent(%)	Yes	634.00(64.60)	209.00(62.80)	425.00(65.60)	0.767	0.381
	No	347.00(35.40)	124.00(37.20)	223.00(34.40)		
Hypertensive(%)	Yes	326.00(33.20)	132.00(39.60)	194.00(29.90)	9.330	0.002**
	No	655.00(66.80)	201.00(60.40)	454.00(70.10)		
Stroke (%)	Yes	116.00(11.80)	64.00(19.20)	52.00(8.00)	26.438	0.000**
	No	865.00(88.20)	269.00(80.80)	596.00(92.00)		
DR(%)	Yes	287.00(29.30)	125.00(37.50)	162.00(25.00)	16.706	0.000**
	No	694.00(70.70)	208.00(62.50)	486.00(75.00)		
DPN(%)	Yes	639.00(65.10)	235.00(70.60)	404.00(62.30)	6.553	0.000**
	No	342.00(34.90)	98.00(29.40)	244.00(37.70)		
DPVD(%)	Yes	193.00(19.70)	87.00(26.10)	106.00(16.40)	13.281	0.000**
	No	788.00(80.30)	246.00(73.90)	542.00(83.60)		
Laboratory biochemical						
HbA1c(%)		10.30(8.00, 12.80)	12.10(10.00, 14.10)	9.70(7.40, 11.60)	-11.438	0.000*
FBG(mmol/L)		10.60(8.60, 12.80)	11.20(9.40, 13.50)	10.40(8.10, 12.50)	-5.880	0.000*
TG(mmol/L)		1.70(1.10, 2.50)	1.70(1.10, 2.50)	1.70(1.10, 2.50)	-0.740	0.459
TC(mmol/L)		4.60(3.90, 5.40)	4.70(4.00, 5.40)	4.50(3.90, 5.30)	-1.465	0.143
HDL-C(mmol/L)		1.10(0.90, 1.30)	1.10(0.90, 1.30)	1.10(0.90, 1.30)	-0.470	0.638
LDL-C(mmol/L)		3.40(2.80, 4.00)	3.60(3.10, 4.10)	3.30(2.70, 3.90)	-6.601	0.000**
Serum C-peptide (pmol/L)		691.80 (471.50, 930.50)	713.60 (478.80, 955.70)	684.80 (463.90, 923.70)	-1.161	0.246
2 h Serum C-peptide (pmol/L)		1432.00 (866.60, 2023.00)	1499.00 (932.30, 2124.50)	1391.50 (854.73, 200.50)	-1.425	0.154
ALB(g/L)		40.30(38.00, 42.50)	40.30(37.90, 42.40)	40.30(38.00, 42.70)	-0.769	0.442
Scr(umol/L)		69.00(58.50, 77.00)	68.00(58.00, 78.00)	69.00(59.00, 77.00)	-0.408	0.684
UA(umol/L)		327.50 (278.00, 365.00)	327.50 (280.50, 362.00)	327.50 (274.30, 365.80)	-0.268	0.789
BUN(mmol/L)		5.20(4.80, 6.10)	5.20(4.20, 6.10)	5.20(4.30, 6.10)	-0.234	0.815
NLR		3.20(2.70, 4.00)	3.50(3.00, 4.20)	3.10(2.30, 3.90)	-7.427	0.000**
PLR		102.10 (79.60, 132.90)	106.00 (84.00, 135.80)	99.90 (77.90, 130.30)	-2.215	0.027*
MPV		11.60(10.80, 12.50)	11.60(10.70, 12.50)	11.70(10.80, 12.40)	-0.681	0.496
25(OH)D(ug/L)		20.60(16.70, 24.90)	19.00(14.80, 24.40)	21.30(17.80, 25.10)	-4.880	0.000**

*, *P*<0.05, **, *P*<0.01.

### Predictive model performance

To uncover the specific independent factors contributing to the occurrence of MAU in individuals diagnosed with T2DM, further research is essential. Sixteen factors, identified as statistically significant in the univariate analysis, were employed as independent variables. The presence or absence of MAU was designated as the dependent variable (assigned as 0 = no, 1 = yes). The criterion for entering the model was set at *P* < 0.05, utilizing the conditional forward method for logistic regression analysis. The results indicated (Table 2) that age, BMI, Stroke, DR, DPVD, LDL-C, NLR, HbA1c, and 25(OH)D independently influenced the progression of T2DM to MAU. These nine variables were then used as inputs for both Random Forest and BP neural network algorithms.

**Table 2:** Predictors Correlated with the Logistic Regression Model

Variable	OR (95% CI)	*P* value
Age	1.022(1.01–1.04)	**0.003**
BMI	1.08(1.033–1.129)	**0.001**
Stroke	1.892(1.202–2.979)	**0.006**
DR	1.454(1.051–2.011)	**0.024**
DPVD	1.543(1.063–2.239)	**0.023**
HbA1c	1.254(1.19–1.322)	**0.000**
LDL-C	1.379(1.148–1.656)	**0.001**
NLR	1.117(1.012–1.219)	**0.027**
25(OH)D	0.956(0.933–0.979)	**0.000**

**Fig. 2:**
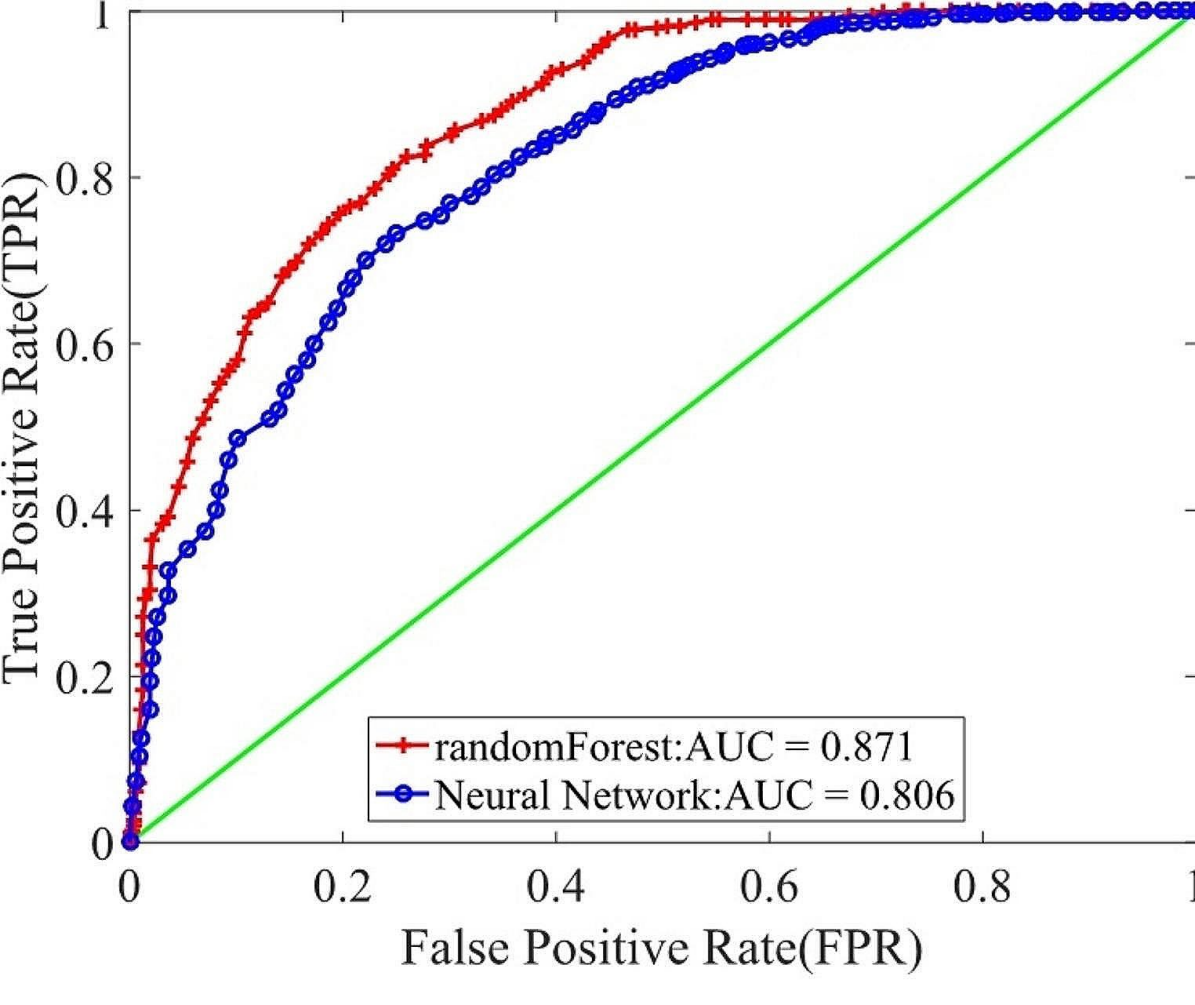
ROC curve and AUC value for Random Forest and BP Neural Network Predict MAU Outcomes in T2DM

Two machine learning models, Random Forest and Back Propagation (BP) Neural Network, were constructed separately. The performance evaluation results are outlined in Table 3, while the ROC curves of Random Forest and BP Neural Network are depicted in Fig. 2. The analysis indicates that the Random Forest algorithm yields higher values in key performance metrics such as the Area Under the ROC Curve (AUC), accuracy, recall rate, specificity, and F1 score, when predicting MAU in T2DM patients. Consequently, the Random Forest model demonstrates superior overall performance compared to the BP Neural Network. As a result, Random Forest was chosen for further prediction and analysis.

**Table 3:** Model Performance Evaluation of Machine Learning Algorithms

Machine Learning Algorithms	AUC	Accuracy	Precision	Recall	Specificity	F1 score
Random forest	0.87	0.80	0.79	0.84	0.76	0.81
BP neural network	0.80	0.74	0.77	0.74	0.73	0.74

### Visualization of feature importance

**Fig. 3:**
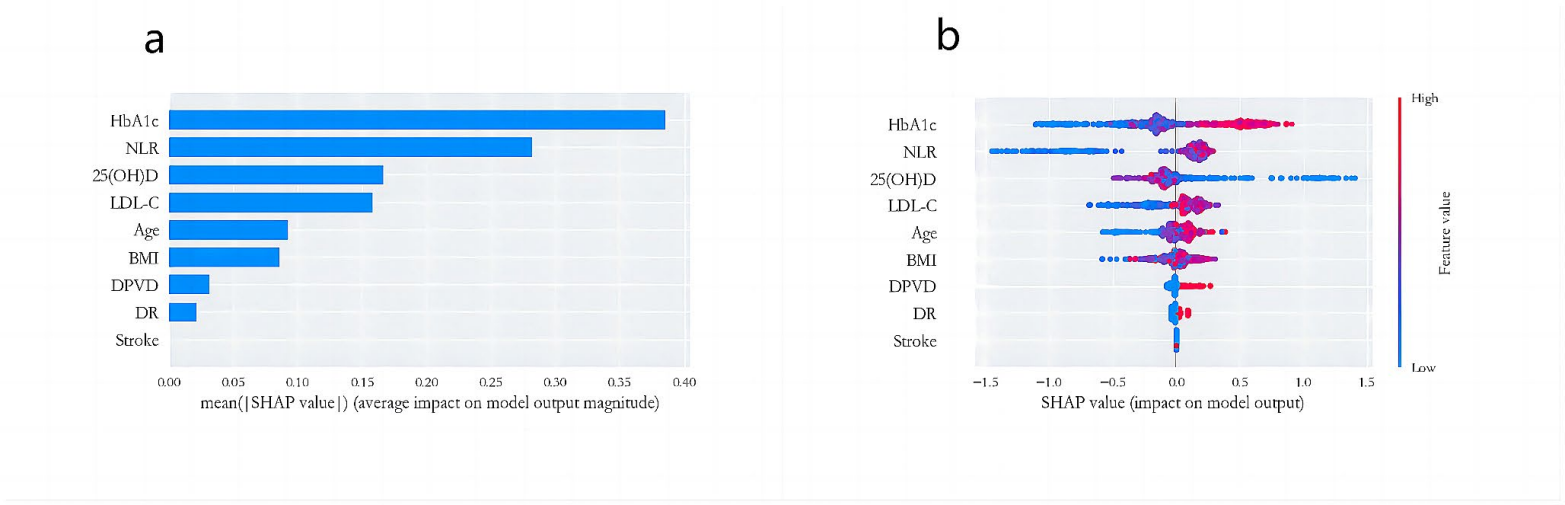
SHAP Analysis for Ranking the Importance of Risk Factors. **a**, Importance Matrix Plot: This depicts the significance of each variable in predicting MAU risk in patients with T2DM. **b**, SHAP Summary Plot: Illustrating the top 9 clinical features of the predictive model, each dot represents a patient per feature and is color-coded based on attribution value (red for higher, blue for lower). The dot’s position indicates whether the feature increases (right) or decreases (left) in the risk prediction. The distance from 0 reflects the contribution to the prediction, with greater distances signifying greater impact

Given the comprehensive performance of the Random Forest model, it was selected for analyzing the influencing factors of MAU in T2DM patients. The visualization of this analysis is presented in Fig. 3, illustrating the contribution of the top 9 features at an overall level. According to the SHAP plot, HbA1c emerges as the most significant factor impacting the model. Moreover, higher HbA1c values correspond to higher Shapley values, indicating an increased likelihood of MAU. Additionally, NLR stands out as another influential feature, where higher values correlate with a higher probability of MAU. Conversely, 25(OH)D exhibits an inverse relationship, with higher values associated with a lower probability of MAU, suggesting its protective role. Furthermore, it was observed that stroke contributes minimally to the model and has little impact on the occurrence of MAU.

## Discussion

The incidence of DKD on a global scale exhibits an upward trend annually, with a multifaceted and diverse array of risk factors contributing to its development. The prediction of proteinuria incidence in patients with DKD is a significant challenge, particularly in those individuals who are in the early stages of the disease. Traditionally, prediction models for MAU in T2DM patients have predominantly relied on logistic regression methods. However, the limitations of traditional data analysis methods, particularly in handling high-dimensional data, have led researchers to explore the advantages of machine learning algorithms. In this context, both Random Forest and BP neural network, employed in this study, are well-suited for classification and regression problems. In the study, risk prediction models for the occurrence of MAU in patients with T2DM were developed using machine learnings. The evaluation metrics, including AUC value, accuracy, precision recall, specificity and F1 score, indicated that the Random Forest model outperformed the BP neural network, demonstrating higher discriminative ability. Compared to the BP neural network, Random Forest offers several advantages: Random Forest comprises a collection of independent decision tree algorithms, endowing it with robust noise anti-interference capabilities when encountering outliers and missing values in the data. This characteristic makes it less susceptible to overfitting [15]. Random Forest exhibits high robustness and generalization ability, whether dealing with a large number of continuous variables or categorical variables.Numerous studies have demonstrated the efficacy of Random Forest in disease prediction, highlighting its practicality and suitability for various medical applications [16, 17].

Development models offer several advantages. As far as current knowledge extends, this study marks the first endeavor to construct a risk prediction model for MAU in individuals with T2DM. The model integrates Random Forests and BP neural network. Secondly, addressing challenges related to sparse sample characterization and shifted decision boundaries caused by unbalanced data, we employed SMOTE technology to balance the dataset. Thirdly, we enhance our model evaluation process by incorporating 5-fold cross-validation. This technique bolsters the robustness and reliability of model evaluation by iteratively partitioning the training set into five subsets, each serving as both a training and test set. By averaging the performance metrics across these iterations, we obtain a more comprehensive understanding of the model’s predictive power. Consequently, our evaluation confirms that Random Forest Random Forest is the better model. Additionally, to enhance interpretability of model predictions, we utilized the SHAP method to elucidate the relationship between input features and model output. The SHAP diagram was employed to rank the influencing factors, facilitating clinicians’ intuitive comprehension and bolstering the model’s clinical applicability. Our findings reveal that key indicators extracted objectively from real clinical data exhibit high correlation with the occurrence of MAU.

Similar to prior research, this study underscores the significance of HbA1c in the context of MAU, with its importance ranking highest. The findings indicate that each 1% increase in HbA1c level corresponds to a 25.4% increase in the risk of developing MAU. HbA1c reflects the average blood glucose level over the past 2–3 months, yet it can be influenced by clinical conditions such as liver and kidney diseases, and it does not promptly capture short-term glucose fluctuations. Time in Range (TIR) is a valuable indicator in continuous glucose monitoring (CGM) [18], but its application in clinical settings is limited due to scope and accuracy constraints. Despite this, HbA1c remains the primary choice for assessing glucose management in diabetes patients [19], and the Global Renal Prognosis Improvement Organization recommends maintaining HbA1c levels at 7% as an effective strategy for reducing microvascular complications in diabetes.

Previous research has established that systemic inflammation serves as a predictor for DKD, with NLR recognized as a valid indicator of systemic inflammation [20]; This current study corroborates these findings, ranking NLR as the second most significant characteristic. Another study [21] highlighted that individuals with MAU exhibit higher NLR levels compared to those without MAU, aligning with the observations in the present study. The hyperfiltration of kidneys and the onset of proteinuria can result from the activation and release of inflammatory agents triggered by metabolic abnormalities [22], leading to direct damage to the vascular endothelium and glomerular basement membrane. NLR emerges as a potentially beneficial diagnostic tool for proteinuria due to its simplicity, cost-effectiveness, and accessibility, with stable results. It reflects the balance between inflammatory regulatory lymphocytes and inflammatory activator neutrophils. NLR has the potential to contribute to proteinuria diagnosis, and its simplicity, low cost, and widespread availability make it an appealing option for testing in both community settings and outpatient clinics.

The prevalence of vitamin D deficiency in DM is a notable concern, and its role in diabetes and associated complications has been extensively studied. Research by Qi Lu et al. [23] demonstrated an association between higher 25(OH)D concentrations and reduced all-cause and cardiovascular disease mortality in pre-diabetic individuals. Several studies in recent years have suggested that 25(OH)D is often deficient in patients with T2DM, and low levels of this vitamin are linked to an increased risk of developing T2DM [24]. Furthermore, inadequate or deficient levels of 25(OH)D have been implicated in an elevated incidence of DKD in patients with T2DM, particularly in the elderly [25]. the mean 25(OH)D level in patients with DKD was found to be lower than in the group without DKD, indicating an association between reduced 25(OH)D levels and the progression of DKD. This observation underscores the potential significance of monitoring vitamin D levels in the context of diabetic complications. However, it’s worth noting that there are differing conclusions in the literature. For example, a study by Lian Engelen [26] concluded that the reduction of 25(OH)D was not associated with the development of MAU in diabetic patients. This discrepancy may be attributed to factors such as the age of the study population, where age-related renal changes could impact vitamin metabolism. While 25(OH)D is considered a plausible marker for vitamin D status and a potential predictor for DKD [27], it’s important to acknowledge variations in study findings. For instance, a cross-sectional study in China revealed a nonlinear relationship between serum 25(OH)D and the albuminuria-to-creatinine ratio in T2DM, showing a negative correlation when 25(OH)D was less than 25 nmol/L. Recommendations for vitamin D supplementation, such as a minimum of 1,500 to 2,000 IU/day, have been proposed to consistently elevate blood 25(OH)D levels above 30 ng/ml [28]. Data from the National Health and Nutrition Examination Survey (NHANES) in the USA (2007–2010) indicate that vitamin D supplementation can effectively increase serum 25(OH)D concentrations in humans [29].

Moreover, multifactorial analysis results identified age, BMI, LDL-C, DR, and DPVD as independent risk factors for MAU. These findings align partially with existing studies [30]. The aging process may lead to degenerative changes in organ tissues, including vascular endothelial dysfunction, contributing significantly to MAU formation. Therefore, emphasizing MAU detection in the elderly is crucial for the early identification of renal damage. BMI serves as a controllable risk factor, and meta-analysis results indicate that both overweight and obesity elevate the risk of DKD [31]. LDL-C is recognized as another crucial factor in early DKD, with previous studies providing a scientific basis for its significance [32]. In diabetes mellitus, elevated LDL-C levels in early DKD patients signify disturbances in glucose metabolism affecting lipid synthesis and slowing catabolism. The study results indicate that LDL-C is an independent risk factor for T2DM patients, with the risk increasing by 37.9% for every 1 mmol/L increase in LDL-C, holding other factors constant. Additionally, MAU is well-established as associated with DR and DPVD, serving as a sensitive marker for cardiovascular disease [10], The study results reaffirm this association, suggesting that individuals with DKD and peripheral vascular disease have a predictive value for early diabetic nephropathy risk.

### Limitations

This study has several limitations. Its retrospective nature makes it susceptible to biases such as selective and recall bias, necessitating additional prospective investigations to validate the observed causal link. Moreover, being conducted in a single center, the study may not be fully representative of the entire Chinese diabetes population, warranting multi-center studies to enhance generalizability. Additionally, factors influencing early nephropathy in T2DM, such as diet, exercise, and socio-economic status, were not investigated in this study. Future research should consider these factors as they play a crucial role in disease development. Researchers in the future may explore additional aspects to further enhance the comprehensiveness of their evaluations.

## Conclusion

In summary, this study successfully identified risk factors associated with MAU in individuals diagnosed with T2DM. By prioritizing intervention on modifiable risk factors and enhancing protective factors, it may be possible to effectively prevent or delay the onset of MAU in patients with T2DM. The Random Forest and BP neural network models both exhibit improved predictive capabilities for MAU in diabetic patients. However, slight variations in results between the models suggest that a combination of multiple models should be considered in practical applications to comprehensively assess predictive performance.

## Data Availability

The datasets used and/or analyzed in the study are available from the corresponding author upon reasonable request. **Corresponding author** Corresponding author: Xiao-Hua Wang, Ph.D., Prof. 350031712@qq.com.
